# A propensity score-matched analysis comparing outpatient and short-stay hospitalization to standard inpatient hospitalization following total ankle arthroplasty

**DOI:** 10.1186/s13018-020-01793-5

**Published:** 2020-07-31

**Authors:** Mark A. Plantz, Alain E. Sherman, Anish R. Kadakia

**Affiliations:** 1 Department of Orthopaedic Surgery, Northwestern University Feinberg School of Medicine, 676 N. Saint Clair St., Suite 1350, Chicago, Cook County, IL 60611 USA; 2 Kellogg School of Management at Northwestern University, 2211 Campus Drive, Evanston, Cook County, IL 60208 USA

**Keywords:** Total ankle arthroplasty, TAA, Short stay, Outpatient, Inpatient, Outcome

## Abstract

**Background:**

Given the trend toward value-based care, there has been increased interest in minimizing hospital length of stay (LOS) after orthopedic procedures. Outpatient total ankle arthroplasty (TAA) has become more popular in recent years; however, research on surgical outcomes of this procedure has been limited. This study sought to employ large sample, propensity score-matched analyses to assess the safety of outpatient and short-stay discharge pathways following TAA.

**Methods:**

The ACS NSQIP database was used to identify 1141 patients who underwent primary and revision TAA between 2007 and 2017. Propensity score matching was used to match patients based on several factors, including age, sex, body mass index (BMI), American Society of Anesthesiologists (ASA) classification, and several comorbidities. The incidence of various 30-day complications was compared between the short and standard LOS groups to assess for any differences in short-term outcomes.

**Results:**

A total of 892 patients were included in the final propensity score-matched analysis, with 446 patients in each group. The short LOS group had a significantly lower rate of medical complications (0.2% vs. 2.5%, *p* = 0.006) and non-home discharge (1.3% vs. 12.1%, *p* < 0.001). There was no significant difference in operative complications (0.4% vs. 1.8%, *p* = 0.107), unplanned readmission (0.4% vs. 1.1%, *p* = 0.451), reoperation (0.2% vs. 0.4%, *p* > 0.999), return to the OR (0.2% vs. 0.9%, *p* = 0.374), or mortality (0.7% vs. 0.0%, *p* > 0.249) between the short and standard LOS groups.

**Conclusions:**

Outpatient and short-stay hospitalization had comparable safety to standard inpatient hospitalization after TAA. Outpatient or short-stay TAA should be considered for patients with low risk of short-term complications.

## Background

As the outcomes of total ankle arthroplasty (TAA) have continued to improve over the last two decades—with the advent of improved third-generation prostheses and refined operative techniques—the number of TAAs performed in the USA has continued to increase [1–3]. TAA has the potential to provide substantial improvement in ankle pain and function in patients with end stage ankle arthritis [4–7]. The benefits of TAA relative to traditional ankle arthrodesis (AA), however, are uncertain and controversial. Some studies have suggested that TAA may provide better long-term pain relief and patient-perceived postoperative function relative to fusion, although others have argued that the two procedures have similar long-term outcomes [8–12]. Nonetheless, ankle replacement has continued to rise in popularity, and the number of providers offering TAA has increased substantially in the last decade [2].

Value-based care and cost minimization continue to be an important factor in modern healthcare delivery. The average postoperative length of stay (LOS) is often used as an indicator of efficiency and value-based care in orthopedic surgery. A substantial percentage of the costs associated with elective joint replacement surgery comes from inpatient hospitalization and subsequent readmissions and/or reoperations [13]. Readmission, in particular, has become an important quality metric tied to reimbursement [14]. Minimizing hospitalization after surgical procedures, where appropriate, has therefore been of increasing importance.

Within the last decade, there has been substantial interest in performing various elective orthopedic procedures in outpatient or short-stay inpatient settings in order to reduce costs and improve patient satisfaction. Much of the research efforts have been dedicated to total hip and knee arthroplasty, given the high disease burden and volume of these procedures performed annually. Several studies have demonstrated substantial cost savings for outpatient and/or short-stay inpatient hospitalization after total knee and hip arthroplasty [15, 16]. However, the findings regarding the risk of short-term complications in patients with shorter hospital stays have been conflicting. Some studies have suggested that outpatient hip and knee arthroplasty may have higher rates of short-term complications relative to inpatient procedures, while others have suggested that there is not a substantial difference in short-term outcome measures between the two groups [17–19]. Many of these studies have utilized national databases that provide large enough sample sizes to investigate even the rarest of complications.

Although shorter hospital stays have been investigated for hip and knee arthroplasty, the same cannot be said for TAA, which presents its own unique challenges. Three separate studies have described outpatient TAA with excellent short-term outcomes and minimal complications [20–22]. However, these studies all shared the limitation of a small sample size (*N* < 100), making it difficult to quantify short-term outcomes, particularly rare complications. A population database study of 591 patients undergoing TAA between 2006 and 2015 demonstrated that outpatient TAA did not yield higher rates of short-term complications relative to standard inpatient TAA [23]. However, this study used unadjusted direct comparisons between the outpatient and inpatient cohorts, and the inpatient cohort had substantially more operative risk factors, including older age and higher comorbidity burden, namely, diabetes [23]. Therefore, directly comparing these populations without adjustment likely introduced confounding and sample bias.

To the authors’ knowledge, there have not been any large sample, propensity score-matched analyses to assess outcomes of outpatient or short-stay hospitalization following TAA, while controlling for comorbidities and other confounding variables. Although decreasing LOS has the potential to increase quality of care, minimize costs, and improve patient satisfaction, careful consideration must be taken to avoid complications and readmission. The present study sought to examine risk factors for various short-term complications following TAA and used propensity score matching to compare the risks of these complications in outpatient and short-stay inpatient hospitalization versus standard inpatient hospitalization.

## Methods

### Sample selection

The American College of Surgeon’s National Surgery Quality Improvement Program (ACS NSQIP) database was queried to identify all patients undergoing TAA between January 1, 2007 and December 31, 2017. Both primary and revision procedures were considered using Current Procedural Terminology (CPT) codes 27702 and 27703. The ACS NSQIP database reports de-identified patient data and has been deemed HIPPA compliant. The Institutional Review Board (IRB) of Northwestern University approved this study as a retrospective cohort study.

The data provided in the ACS NSQIP database have been extensively investigated in various surgical fields, often to determine the incidence of short-term complications, identify risk factors for adverse short-term outcomes, and risk stratify patients for various procedures [24–27]. ACS NSQIP reports over 150 variables, including patient demographics, comorbidities, lifestyle factors, preoperative laboratory values, operative variables, 30-day operative and medical complications, and 30-day disposition outcomes (e.g., return to OR, reoperation, and readmission). Given the substantial number of cases, the database is ideal for assessing low incidence complications after various procedures. ACS NSQIP has been shown to have excellent validity, reliability, and a consistently low rate of reporting error [28–30]. Data sampling methodologies at participating institutions are routinely monitored, and interrater reliability audits are regularly performed to ensure data accuracy.

### Measures

The total number of TAAs performed was identified for each year between 2011 and 2017. A one-way analysis of variance (ANOVA) with Games-Howell post-hoc test was used to compare the mean postoperative LOS after TAA for each year. For analysis of outcomes, only data between 2011 and 2017 were considered because 2011 was the first year that ACS NSQIP began reporting certain 30-day outcomes, including unplanned readmission, reoperation, and return to the operating room.

Prior to propensity score matching, several patient variables—sex, age group, race/ethnicity, BMI classification, ASA classification, and comorbidities (diabetes, smoking, COPD, congestive heart failure, hypertension, dialysis, chronic steroid use)—were compared for patients with outpatient or short-stay hospitalization (LOS ≤ 1 day) versus standard inpatient hospitalization (LOS > 1 day). Pearson’s chi-squared test or Fisher’s exact test, where appropriate, were used to compare these categorical variables and various 30-day outcome measures between the two samples, including non-home discharge, mortality, return to the OR, readmission, reoperation, operative complications (surgical site infection, dehiscence, bleeding), and medical complications (wound infection, pneumonia, reintubation, failure to wean intubation, pulmonary embolism, renal insufficiency, renal failure, urinary tract infection, cerebral vascular accident, cardiac arrest, myocardial infarction, deep venous thrombosis, systemic sepsis, septic shock).

### Propensity score matching and statistical analysis

Propensity score matching was used to control for the differences in both modifiable and non-modifiable risk factors between the two disposition groups (LOS ≤ 1 day and LOS > 1 day). Specifically, patients from the two groups were paired in a 1:1 manner using a balanced, nearest neighbor approach based on the following variables: sex, age, BMI, ASA classification, and comorbidities (diabetes, smoking, COPD, CHF, hypertension, and chronic steroid use). All categorical variables (sex, ASA classification, and comorbidities) were matched exactly between the two groups. Continuous variables (age and BMI) were matched with a pre-defined tolerance of ± 10 years and ± 5 kg/m^2^, respectively. Additionally, age was divided into five groups: under 50, between 50 and 59, between 60 and 69, between 70 and 79, and 80 and over. BMI was divided into six groups: underweight (BMI < 18.5), normal (BMI 18.5 to 24.9), overweight (BMI 25.0 to 29.9), obesity class I (BMI 30.0 to 34.9), obesity class II (BMI 35.0 to 39.9), and obesity class III (BMI ≥ 40.0). After propensity score matching, Pearson’s chi-squared test and, where appropriate, Fisher’s exact test were used to compare the rate of the patient and operative variables described previously to ensure these factors were statistically equivalent between the two groups. Lastly, the same statistical tests described previously were used to compare the rate of the 30-day complications between the matched groups. All statistical analyses were completed using IBM SPSS Version 24 (IBM Corp., Armonk, NY). The criterion for statistical significance was set at *p* ≤ 0.05.

## Results

**Fig. 1:**
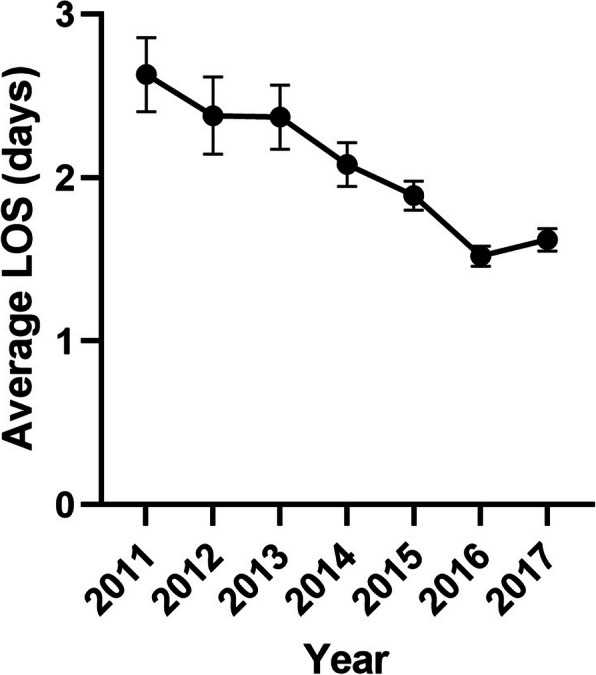
Average length of stay following total ankle arthroplasty annually between 2011 and 2017, calculated using data from the ACS NSQIP database cohort. Error bars report the standard error (SE)

A total of 1231 TAA procedures (106 revision TAA and 1125 primary TAA) were identified in the ACS NSQIP database between 2007 and 2017. The total number of procedures reported in the ACS NSQIP database increased significantly over the decade (4 in 2007 to 286 in 2017). Additionally, the average postoperative LOS following TAA surgery decreased between 2011 and 2017 (Fig. 1). In the final analysis, 1141 TAA procedures (106 revision TAA and 1035 primary TAA) were included from years 2011 through 2017. Data prior to this period were excluded because 30-day outcomes were not reported until 2011. The majority of patients were between 60 and 79 years old (63.9%), white (74.7%), overweight or obese (85.8%), and had an ASA score of 2 or 3 (93.9%). A substantial minority had one or more reported comorbidity (12.4% with diabetes, 8.1% with active smoking, and 5.1% with chronic steroid use).

Patients with shorter LOS after TAA (LOS ≤ 1) tended to be younger and healthier overall at baseline (Table 1). This group had more patients between ages 50 and 59 (26.0% vs. 20.9%, *p* = 0.043), a lower prevalence of diabetes (10.5% vs. 14.3%, *p* = 0.050), and fewer individuals with an ASA class of 3 (32.2% vs. 43.4%, *p <* 0.001). However, the short LOS group tended to have higher rates of active smoking (10.0% vs. 6.2%, *p* = 0.020).

**Table 1:** Patient demographics and risk factors in short and standard LOS groups

	LOS ≤ 1 day (***n*** = 562)		LOS > 1 day (***n*** = 579)		***p***
Revision TAA	8.7%	(49/562)	9.8%	(57/579)	0.513
Sex					
Male	58.4%	(328/562)	45.1%	(261/579)	< 0.001*
Female	41.6%	(234/562)	54.9%	(318/579)	
Race/ethnicity					
Asian	0.9%	(5/562)	0.3%	(2/579)	0.281
Black or African American	2.1%	(12/562)	3.6%	(21/579)	0.133
Hispanic	2.7%	(15/562)	2.9%	(17/579)	0.785
White	83.8%	(471/562)	65.8%	(381/579)	< 0.001*
Other	0.4%	(2/562)	0.3%	(2/579)	> 0.999
Unknown/not reported	10.1%	(57/562)	26.9%	(156/579)	< 0.001*
Age (years)					
Under 50	8.0%	(45/562)	7.9%	(46/579)	0.969
Between 50 and 59	26.0%	(146/562)	20.9%	(121/579)	0.043*
Between 60 and 69	38.8%	(218/562)	38.5%	(223/579)	0.924
Between 70 and 79	23.8%	(134/562)	26.8%	(155/579)	0.256
80 and over	3.4%	(19/562)	5.9%	(34/579)	0.046*
BMI (kg/m^2^)					
Underweight	0.5%	(3/562)	1.0%	(6/579)	0.507
Normal	11.9%	(67/562)	14.9%	(86/579)	0.146
Overweight	39.1%	(220/562)	31.8%	(184/579)	0.009*
Obese class I	26.9%	(151/562)	28.2%	(163/579)	0.627
Obese class II	14.6%	(82/562)	16.9%	(98/579)	0.279
Obese class III	6.9%	(39/562)	7.3%	(42/579)	0.836
Comorbidities					
Diabetes	10.5%	(59/562)	14.3%	(83/579)	0.050*
Smoker	10.0%	(56/562)	6.2%	(36/579)	0.020*
COPD	2.3%	(13/562)	2.4%	(14/579)	0.907
Congestive heart failure	0.2%	(1/562)	0.2%	(1/579)	> 0.999
Hypertension	55.0%	(309/562)	56.1%	(325/579)	0.696
Dialysis	0.0%	(0/562)	0.3%	(2/579)	0.500
Chronic steroid use	4.1%	(23/562)	6.0%	(35/579)	0.133
ASA class					
Class 1 (no disturbance)	6.0%	(34/562)	3.4%	(20/579)	0.039*
Class 2 (mild disturbance)	60.5%	(340/562)	51.6%	(299/579)	0.003*
Class 3 (severe disturbance)	32.2%	(181/562)	43.4%	(251/579)	< 0.001*
Class 4+ (life threatening)	1.3%	(7/562)	1.6%	(9/579)	0.657

* indicates statistical significance (*p* < 0.05)

The unmatched data demonstrated that patients with shorter LOS after TAA had better outcomes with fewer complications relative to the standard LOS group (Table 2). Specifically, the short LOS group demonstrated lower rates of non-home discharge (1.4% vs. 14.5%, *p* < 0.001), operative complications (0.4% vs. 2.1%, *p* = 0.013), and medical complications (0.4% vs. 2.1%, *p* = 0.013), particularly urinary tract infections (0.0% vs. 1.4%, *p* = 0.015).

**Table 2:** Short-term outcomes and complications in short and standard LOS groups

	LOS ≤ 1 day (***n*** = 562)		LOS > 1 day (***n*** = 579)		***p***
Non-home discharge	1.4%	(8/562)	14.5%	(84/579)	< 0.001*
Mortality	0.5%	(3/562)	0.0%	(0/579)	0.119
Return to OR	0.2%	(1/562)	1.0%	(6/579)	0.124
Readmission	0.5%	(3/562)	1.4%	(8/579)	0.225
Reoperation	0.2%	(1/562)	0.4%	(2/579)	> 0.999
Operative complications					
Overall^a^	0.4%	(2/562)	2.1%	(12/579)	0.013*
Surgical site infection	0.2%	(1/562)	1.0%	(6/579)	0.124
Dehiscence	0.0%	(0/562)	0.5%	(3/579)	0.250
Bleeding requiring transfusion	0.2%	(1/562)	0.9%	(5/579)	0.218
Medical complications					
Overall^a^	0.4%	(2/562)	2.1%	(12/579)	0.013*
Wound Infection	0.0%	(0/562)	0.2%	(1/579)	> 0.999
Pneumonia	0.2%	(1/562)	0.2%	(1/579)	> 0.999
Reintubation	0.0%	(0/562)	0.2%	(1/579)	> 0.999
Failure to wean intubation	0.0%	(0/562)	0.0%	(0/579)	–
Pulmonary embolism	0.4%	(2/562)	0.0%	(0/579)	0.242
Renal insufficiency	0.0%	(0/562)	0.0%	(0/579)	–
Renal failure	0.0%	(0/562)	0.0%	(0/579)	–
Urinary tract infection	0.0%	(0/562)	1.4%	(8/579)	0.015*
Cerebrovascular accident	0.0%	(0/562)	0.0%	(0/579)	–
Cardiac arrest	0.0%	(0/562)	0.0%	(0/579)	–
Myocardial infarction	0.0%	(0/562)	0.2%	(1/579)	> 0.999
Deep venous thromboembolism	0.2%	(1/562)	0.3%	(2/579)	> 0.999
Systemic sepsis	0.2%	(1/562)	0.0%	(0/579)	0.493
Septic shock	0.0%	(0/562)	0.0%	(0/579)	–

* indicates statistical significance (*p* < 0.05)

^a^The overall percentages may not equal the sum of the individual percentages, because some patients had multiple operative or medical complications

Table 3 compares the same patient and operative variables summarized in Table 1 after propensity score matching. A total of 892 patients were included in this analysis, with 446 patients per group. After matching, there were no statistically significant differences in sex, age group, BMI group, comorbidities (diabetes, active smoking, COPD, CHF, hypertension, dialysis, and chronic steroid use), ASA classification, or primary vs. revision TAA (*p*s > 0.05) between the two groups. Table 4 compares the rate of various 30-day complications and outcome measures between the matched groups. The short LOS group had a significantly lower rate of medical complications (0.2% vs. 2.5%, *p* = 0.006) and non-home discharge (1.3% vs. 12.1%, *p* < 0.001) relative to the standard LOS group. There was no significant difference in operative complications (0.4% vs. 1.8%, *p* = 0.107), unplanned readmission (0.4% vs. 1.1%, *p* = 0.451), reoperation (0.2% vs. 0.4%, *p* > 0.999), return to the OR (0.2% vs. 0.9%, *p* = 0.374), or mortality (0.7% vs. 0.0%, *p* > 0.249) between the short and standard LOS groups.

**Table 3:** Patient demographics and risk factors in short and standard LOS groups after propensity score matching

	LOS ≤ 1 day (***n*** = 446)		LOS > 1 day (***n*** = 446)		***p***
Revision TAA	10.5%	(47/446)	11.7%	(47/446)	0.594
Sex					
Male	52.2%	(233/446)	52.2%	(233/446)	> 0.999
Female	47.8%	(213/446)	47.8%	(213/446)	
Race/ethnicity					
Asian	0.9%	(4/446)	0.4%	(2/446)	0.686
Black or African American	2.0%	(9/446)	3.4%	(15/446)	0.214
Hispanic	2.5%	(11/446)	2.5%	(11/446)	> 0.999
White	84.1%	(375/446)	65.7%	(293/446)	< 0.001*
Other	0.2%	(1/446)	0.4%	(2/446)	> 0.999
Unknown/not reported	10.3%	(46/446)	27.6%	(123/446)	< 0.001*
Age (years)					
Under 50	7.8%	(35/446)	7.0%	(31/446)	0.609
Between 50 and 59	26.0%	(116/446)	22.9%	(102/446)	0.275
Between 60 and 69	39.5%	(176/446)	37.7%	(168/446)	0.582
Between 70 and 79	23.1%	(103/446)	26.5%	(118/446)	0.245
80 and over	3.6%	(16/446)	6.1%	(27/446)	0.086
BMI (kg/m^2^)					
Underweight	0.4%	(2/446)	1.1%	(5/446)	0.451
Normal	12.1%	(54/446)	15.5%	(69/446)	0.145
Overweight	37.6%	(168/446)	35.2%	(157/446)	0.444
Obese class I	26.5%	(118/446)	27.4%	(122/446)	0.763
Obese class II	15.9%	(71/446)	16.4%	(73/446)	0.856
Obese class III	7.4%	(33/446)	4.5%	(20/446)	0.066
Comorbidities					
Diabetes	9.6%	(43/446)	9.6%	(43/446)	> 0.999
Smoker	6.3%	(28/446)	6.3%	(28/446)	> 0.999
COPD	0.7%	(3/446)	0.7%	(3/446)	> 0.999
Congestive heart failure	0.0%	(0/446)	0.0%	(0/446)	> 0.999
Hypertension	52.7%	(235/446)	52.7%	(235/446)	> 0.999
Dialysis	0.0%	(0/446)	0.0%	(0/446)	> 0.999
Chronic steroid use	3.6%	(16/446)	3.6%	(16/446)	> 0.999
ASA class					
Class 1 (no disturbance)	4.5%	(20/446)	4.5%	(20/446)	> 0.999
Class 2 (mild disturbance)	57.8%	(258/446)	57.8%	(258/446)	> 0.999
Class 3 (severe disturbance)	37.2%	(166/446)	37.2%	(166/446)	> 0.999
Class 4+ (life threatening)	0.4%	(2/446)	0.4%	(2/446)	> 0.999

* indicates statistical significance (*p* < 0.05)

**Table 4:** Short-term outcomes and complications in short and standard LOS groups after propensity score matching

	LOS ≤ 1 day (***n*** = 446)		LOS > 1 day (***n*** = 446)		***p***
Non-home discharge	1.3%	(6/446)	12.1%	(54/446)	< 0.001*
Mortality	0.7%	(3/446)	0.0%	(0/446)	0.249
Return to OR	0.2%	(1/446)	0.9%	(4/446)	0.374
Readmission	0.4%	(2/446)	1.1%	(5/446)	0.451
Reoperation	0.2%	(1/446)	0.4%	(2/446)	> 0.999
Operative complications					
Overall^a^	0.4%	(2/446)	1.8%	(8/446)	0.107
Surgical site infection	0.2%	(1/446)	0.7%	(3/446)	0.624
Dehiscence	0.0%	(0/446)	0.4%	(2/446)	0.499
Bleeding requiring transfusion	0.2%	(1/446)	0.9%	(4/446)	0.374
Medical complications					
Overall^a^	0.2%	(1/446)	2.5%	(11/446)	0.006*
Wound Infection	0.0%	(0/446)	0.2%	(1/446)	> 0.999
Pneumonia	0.0%	(0/446)	0.2%	(1/446)	> 0.999
Reintubation	0.0%	(0/446)	0.2%	(1/446)	> 0.999
Failure to wean intubation	0.0%	(0/446)	0.0%	(0/446)	–
Pulmonary embolism	0.2%	(1/446)	0.0%	(0/446)	> 0.999
Renal insufficiency	0.0%	(0/446)	0.0%	(0/446)	–
Renal failure	0.0%	(0/446)	0.0%	(0/446)	–
Urinary tract infection	0.0%	(0/446)	1.6%	(7/446)	0.031*
Cerebrovascular accident	0.0%	(0/446)	0.0%	(0/446)	–
Cardiac arrest	0.0%	(0/446)	0.0%	(0/446)	–
Myocardial infarction	0.0%	(0/446)	0.2%	(1/446)	> 0.999
Deep venous thromboembolism	0.0%	(0/446)	0.4%	(2/446)	> 0.499
Systemic sepsis	0.0%	(0/446)	0.0%	(0/446)	–
Septic shock	0.0%	(0/446)	0.0%	(0/446)	–

* indicates statistical significance (*p* < 0.05)

^a^The overall percentages may not equal the sum of the individual percentages, because some patients had multiple operative or medical complications

## Discussion

The number of TAA procedures performed in the USA has been increasing given improvements in prostheses, an aging population with a high burden of end-stage arthritis, and increasing provider experience with TAA [1–3]. Providing value-based care and optimizing the associated costs of total ankle replacement are, therefore, of significant importance. Additionally, as the average postoperative LOS after TAA continues to decrease, as demonstrated in our results, the potential risk of short-term complications needs to be investigated thoroughly to ensure patient safety.

Although outpatient and short-stay discharge pathways can be used as a tool to decrease costs, these measures must not underscore patient safety. Additionally, these cost savings can be readily countermanded in the event of costly readmissions and reoperations resulting from premature hospital discharge. Notably, the Centers for Medicare & Medicaid Services (CMS) has been utilizing readmission as a surrogate for the quality and efficiency of healthcare delivery, with financial penalties given for above-average risk-adjusted readmission rates for certain conditions [31–33]. Therefore, discharge pathways should be optimized to account for the risk of poor short-term outcomes.

A previous study utilizing the ACS NSQIP database demonstrated that outpatient TAA was not associated with a higher incidence of short-term complications relative to inpatient TAA, although this study did not utilize propensity score matching to account for differences in patient-specific risk factors [23]. Given that the patients with shorter hospitalization after elective TAA were younger and healthier in our population-based sample, it is important to control for these potential confounding risk factors. Our study consists of the largest known sample to date that utilized propensity score matching to specifically address the association of hospital LOS and short-term complications following TAA. The data discussed in this study indicate that outpatient and short-stay discharge pathways do not necessarily pose a greater risk of short-term complications than standard inpatient discharge pathways after TAA. These results suggest that shorter hospital stays can reasonably be considered for appropriate patients.

Shortened hospital stays after elective orthopedic procedures have also been associated with excellent patient satisfaction scores [34–36]. Although patient-reported outcome data are not collected by the ACS NSQIP database, these measures are another variable to consider when selecting a discharge plan. Additionally, shared decision-making would ideally play an important role in this process, with an emphasis on educating patients regarding common complications and signs that warrant readmission. As more data pertaining to short-term outcomes of TAA become available, these discussions can become more meaningful and evidence-based.

Although the findings in this study are promising, there are certain limitations that should be addressed. Of note, the sample size herein is substantially lower than similar studies assessing more common joint replacement procedures (e.g., hip and knee arthroplasty) [19, 37]. The fact that the incidence of short-term complications after elective TAA was so low further warrants the need for studies with larger sample sizes. Substantially, more cases would be required to risk-stratify individual patients and create a precise decision-making algorithm for different discharge pathways. Additionally, the retrospective nature of this study further limits the level of evidence and conclusions that can be drawn.

Furthermore, there are several patient-specific variables that are not captured within the ACS NSQIP database, but would likely influence decisions regarding postoperative length of stay and discharge. For example, regardless of underlying health conditions, the patient’s baseline activity level and overall independence may influence a decision to pursue outpatient surgery. Other factors include the patient’s comfort level and attitude towards outpatient surgery, their eagerness to return to normal activity, their ability to cooperate and successfully participate in the rehabilitation process, and their home support system (e.g., caretakers, family members, spouse). Each individual’s situation is inherently different, and other factors besides the patient’s overall health are certainly important factors that may influence decision-making.

Future investigations, specifically with larger prospective cohorts, are logical next steps to accurately risk stratify patients and determine the ideal candidate for outpatient or short-stay hospitalization after elective TAA.

## Conclusions

The results of this analysis suggest that, after controlling for various risk factors, outpatient and short-stay discharge do not inherently increase the incidence of short-term complications and poor outcomes relative to standard inpatient hospitalization after TAA. The optimal discharge plan following TAA should address both individual and operative risk factors associated with poor short-term outcomes. Short-stay or outpatient TAA can reasonably be considered for low-risk patients. Further investigation, specifically with prospective cohorts, is warranted to improve the current level of evidence regarding this topic.

## Data Availability

The American College of Surgeons National Surgical Quality Improvement Program and the hospitals participating in the ACS NSQIP are the source of the data used; herein, they have not verified and are not responsible for the statistical validity of the data analysis or the conclusions derived by the authors. The datasets generated and/or analyzed during the current study are available in the ACS NSQIP Participant Use Data File repository, https://www.facs.org/quality-programs/acs-nsqip/participant-use.
